#  Investigation of Mental Health in Patients with Medically Unexplained Physical Symptoms‎

**Published:** 2016-01

**Authors:** Frough Riahi, Maryam Izadi-mazidi, Niloufar Khajeddin, Shahriar Nasirzadeh, Fatemeh Shafieian, Ammar Helalinasab, Mozhgan Deilamani

**Affiliations:** 1 ‎ Department of ‎ Psychiatry, Ahvaz Jundishapur University of Medical Sciences, Ahvaz, Iran; 2 Department of Clinical Psychology, Shahed University, Tehran, Iran‎; 3 Specialist in Physical Medicine and Rehabilitation, Imam Khomeini Hospital, Ahvaz, ‎Iran; 4 School of Medicine, Jundishapur University of Medical Sciences, Ahvaz, Iran‎

**Keywords:** Medically Unexplained Symptom, Mental Health, Electro Diagnosis

## Abstract

**Objective: **Medically unexplained symptoms are physical symptoms, which cannot be explained by organic ‎causes. This study aimed to investigate mental health in patients with medically unexplained ‎physical symptoms. ‎

**Method:** One hundred outpatients who were admitted to the Electro Diagnosis Clinic of Imam Khomeini ‎hospital, Ahvaz/Iran, participated in this study. Data were collected using physical examination, ‎paraclinical examinations, and SCL-90-R, and analyzed through multivariate analysis of variance ‎‎ (MANOVA), Chi-square test and Fisher’s exact test. ‎

**Results:** The findings revealed significant differences between clients with medically explained and ‎unexplained symptoms in obsessive compulsive and somatization (p<0.05). Differences in ‎depression, anxiety, phobia, psychosis, aggression and paranoia were not significant (p>0.05).‎

**Conclusion**: The present study suggested an association between some psychological problems and somatic ‎symptoms. Therefore, screening for psychological impairments can improve clinical outcomes.‎

Many patients have a range of persistent bodily complaints that have no identified pathology and ‎cannot be corresponded to any organic disease (1). ‎

These symptoms, which are called medically unexplained symptoms (MUS), are common in all ‎ages and all field of medicine around the world (2). Prevalence of MUS has been reported to be ‎about 25–50% in primary health care and 30 to 70% in specialist care settings (3). Some of the ‎examples of such illnesses are chest pain, headache, backache, fatigue, irritable bowel syndrome, ‎abdominal pain and other gastrointestinal complaints that often coexist in a patient (4). The ‎symptoms are disabling and as severe as those with organic pathology (5). Quality of life of ‎patients is negatively affected by the symptoms and both the patient and the society are ‎burdened by this phenomenon (6).‎

Treatment of patients with MUS can be frustrating (7). Because of unidentified pathology, they are ‎often managed by multiple referrals and treated inadequately (4). They receive therapeutic ‎interventions that are often unhelpful and expensive (8). ‎

There is evidence to suggest that psychological trauma relate to vulnerability to more medical ‎symptoms, and psychological factors play an important role in development of MUS (9). ‎

Between 26 to 58% of patients with MUS have been reported to have co morbid anxiety or/and ‎depression (10, 11, 12) and 37-88.6% of patients have comorbid personality disorder (13, 14, 15, 16, 17). ‎Barsky, Orav and Bates (2005) found significantly higher number of comorbid somatic disorders ‎in these patients than in general primary-care patients (18). Zarghami et al. (2006) and ‎Khajehmougahi and Ahmadi (2008) reported that patients with low back pain experience ‎impairment in some of the dimensions of mental health measured by SCL-90-R (19, 20).‎

Although several studies support the link between psychological problems and MUS, there are ‎some lines 

of evidence that challenge this association; for example, Morriss et al. (1999) did not find an association between depression and the reporting of pain, ‎psychophysiological syndromes and medically unexplained symptoms in patients with chronic ‎fatigue syndrome (21). Jackson (2005) and Nimnuan et al. (2001) did not find an association ‎between anxiety and / or depression with MUS (22). Moreover, some studies have reported higher ‎scores of depression in some somatic syndromes than others (23).‎

Because of these controversial findings, and since the association between medically unexplained ‎symptoms and mental health has received little attention in Iran, the present study was aimed to ‎compare the mental health of patients with medically unexplained physical symptoms and those ‎with a medical diagnosis in Ahvaz, Southwest Iran.‎

## Materials and Method

This study was conducted at the Electro Diagnosis Clinic of Imam Khomeini Hospital affiliated ‎to Jundishapur University of medical sciences in Ahvaz, Iran. The sample of the study consisted ‎of 100 patients (18 years and older) who referred to the Electro Diagnosis Clinic by ‎endocrinologists, orthopedists, neurologists or neurosurgeons. The clinical appraisals by these ‎specialists showed that the patients’ physical complaints had no medical explanations. Therefore, ‎they referred to the center for paraclinical appraisals.‎

Paraclinical appraisals including imaging (CT and MRI), the specific neurologic examination and ‎EMG and NVC were done. Paraclinical assessments were prescribed by a specialist and no ‎additional cost was imposed to the patients.‎

‎After describing the procedures and purposes of the study, informed consent was obtained from the participants. To obtain demographic data about ethnicity, gender, age, level of education and marital status, each participant was asked to complete the Persian version of Symptom Distress Check List (SCL-90-R) as well as a questionnaire. Exclusion criteria included the presence of intense pain, mental retardation (IQ < 70), and dissatisfaction with the cooperation.

The study was approved by the Ethics Committee of the University (No: Eth-105). Data were ‎collected over a period of 12 months from 2014 to 2015.‎

According to the clinical and paraclinical appraisals and specialist diagnosis, patients were ‎divided into two groups: ‎

Group I: Patients with medically unexplained or poorly explained symptoms 

Group II: Patients with medically explained symptoms

***Instruments:***

-      Physical Examinations

-      Paraclinical Tests including Imaging (CT and ‎MRI), EMG, NCV‎

-     Symptom Distress Check List (SCL-90-R): SCL-‎‎90-R is a self-report scale which has ten ‎subscales ‎including obsessive-compulsive (OC), somatization ‎‎(SOM), phobic anxiety ‎‎(PHOB), interpersonal ‎sensitivity (INT), depression (DEP), anxiety (ANX), ‎anger-‎hostility (HOS), psychotism (PYS), paranoid ‎thought (PAR) and extra scale (ES). The ‎scale has ‎a general symptom score. Higher scores represent ‎more problems (24).‎

***Statistical analysis***

We used Kolmogorov–Smirnov‏ ‏test to determine if the data had normal distribution. Data were ‎normally distributed into two groups for total score of SCL-90 and its subscales (all p>0.05). ‎Data were analyzed using multivariate analysis of variance (MANOVA), Chi-square test and ‎Fisher’s exact test. The probability level of 0.05 was accepted as statistically significant. ‎Statistical analyses were carried out using SPSS version 16. ‎

## Results

Among the participants, 19 (19%) had medically unexplained symptoms and 81(81%) had ‎symptoms with identified pathology. ‎

The mean age of the participants was 37.42 years (±14.03) for the patients with MUS and 44.76 ‎years (± 12.45) for patients with clinical diagnosis. There was a significant difference between ‎the two groups in age (t = 2.258, p = 0.02).‎

Frequencies of the participants’ demographic features are listed in Table 1. There was no ‎difference in demographic features including gender, marital status, ethnicity and level of ‎education between the two groups (all p>0.05, Table 1). Nevertheless, difference in age groups ‎was statistically signiﬁcant (p = 0.01). MUS were more prevalent in age groups of 20-29 years ‎and 30-39 years, and less prevalent in age group of <20 years and 60-70 years (Table1). ‎

Table 2 demonstrates physical complaints of the participants.‎‏ ‏Pain in the back, and head and neck ‎were significantly more prevalent in patients with MUS compared to patients without MUS ‎‎(P<0.05).‎

The mental health of the patients with MUS and patients with medical diagnosis was compared ‎using multivariate analysis of variance (MANOVA). There were significant differences between ‎the two groups in obsessive-compulsive (F = 2.67, p = 0.04) and somatization (F = 2.95, p = ‎‎0.03).‎

Differences in aggression, paranoia, depression, anxiety and phobia were not significant at ‎p<0.05. (Table 3)‎

**Table 1:** Frequencies of the participants’ demographic features according to the group

**Feature**		**Patients with medically unexplained symptoms**	**Patients with medically explained symptoms**	**P value**
Age(years)	<20	1(5.26%)	3(3.7%)	0.01
	20-29	5(26.31%)	7(8.64%)	
	30-39	7(36.84%)	14(17.28%)	
	40-49	2(10.52%)	25(30.86%)	
	50-59	3(15.78%)	24(29.62%)	
	60-70	1(5.26%)	8(9.87%)	
Gender	Female	15(78.94%)	53(65.43%)	0.1
	Male	4(21.05%)	28(34.56%)	
Marital status	married	14(73.68%)	70(86.41%)	0.1
	Single	5(26.31%)	6(7.40%)	
	Divorced/widowed	0	3(3.7%)	
Ethnicity	Lor	8 (42.10%)	31(38.27%)	0.7
	Arab	8(42.10%)	27(33.33%)	
	Fars	2(10.52%)	21(25.92%)	
	Kord	0(0)	1(1.23%)	
	Tork	1(5.2%)	1(1.23%)	
Level of Education	Grade school	14 (73.68%)	55(97.90%)	0.8
	12 years/high school	3(15.78%)	15(18.51%)	
	University degree	2(10.52%)	10(12.34%)	

**Table2:** Frequencies of the participants with different pain location according to the group

**Pain locations **	**Backache**	**Pain in ** **Lower extremities**	**Pain in ** **Upper extremities**	**chest**	**Head and neck**
**Patients**					
Patients with MUS	10	17	18	4	8
Patients without MUS	3	14	17	0	3

**Table 3:** Comparison of mental health and its subscales in patients with and without MUS ‎using multivariate analysis of variance (MANOV)

**Mental health and its subscales**	**Patients with MUS**		**Patients without MUS**		**F**_1,98_	***p*** **-value**
	**Mean**	**SD**	**Mean**	**SD**		
SCL-90 General symptom score	182	69.51	174.52	56.79	2.48	0.06
Somatization (SOM)	29.5	11.44	28.67	9.64	2.95	0.03
Obsessive-compulsive (OC)	19.18	8.4	20.55	7.68	2.67	0.048
Interpersonal sensitivity (INT)	16.56	8.37	14.08	5.99	1.37	0.2
Anger/hostility	10.62	4.22	10.64	3.91	1.08	0.3
Anxiety (ANX)	18.87	6.98	17.79	5.94	1.71	0.1
Depression(DEP)	30.5	12.03	29.55	11.36	2.16	0.09
Phobic anxiety (PHOB)	10.62	4.47	9.58	3.45	1.89	0.1
Paranoid thought (PAR)	13.06	4.99	12.44	4.9	1.26	0.2
Psychotism (PYS)	18.87	7.84	18.14	8.29	2.1	0.1

## Discussion

This study was conducted to investigate mental health in patients with medically unexplained physical symptoms and was the first to report mental health in patients with painful somatic symptoms in different parts of body including the head and neck, chest, back, abdomen, hip, lower limb and upper limbs in the Iranian population. Pervious researches (19, 20) in Iran were conducted mainly on patients with low back pain (LBP).

The results revealed significant differences in obsessive-compulsive and somatization between the two groups of patients with and without medically unexplained physical symptoms. Differences in depression, anxiety, phobia, psychosis, aggression and paranoia were not significant. However, Zarghami et al. (2006) found impairment in all dimensions of mental health measured by SCL-90-R in patients with unexplained LBP than in those with well-defined LBP (19). In addition, Khajehmougahi and Ahmadi (2008) (20) reported somatization, psychosis and hostility in patients with LBP.

In contrast to our results, many studies showed depression and anxiety in chronic pain patients with MUS (Carson et al, 2000; Henningsen 2003; Ston et al, 2002; Burton et al, 2011) (25-28). Nevertheless, Jackson (2005), Morriss et al. (1999) and Nimnuan et al. (2001) (21, 22) in line with the current research, did not find any association between anxiety and / or depression with MUS.

We found no significant difference between the two groups in gender, marital status, ethnicity and level of education. Maiden et al. (2002) also reported no association between unexplained symptoms and educational level (29).

But Verhaak (2006) found that patients with MUS are more often female and less educated than the average patients or patients with identified diagnosis (30). Zarghami et al. (2006) reported that MUS are more common in married participants than in single ones (19). 

Prevalence of MUS has been shown to have a wide range of 1.6–70%, 2.4–87%, and 4.6–18%, in the younger, middle (50–65 years) and older (≥65 years) age groups, respectively (31). In the present research, the prevalence of MUS was 78.96%, 15.78%, and 5.26% for the younger, middle-aged (50–65 years) and older age (≥65 years) group, respectively. 

This gap might be somewhat due to the features of the participants, methodological differences in the assessment of psychological factors, lack of agreement in defining MUS, or cultural differences in somatic presentations of psychological problem.

 In this study, we used paraclinical examinations, while a few previous studies had used paraclinical assessments. 

The association between psychological factors and medically unexplained manifestations in the current study may improve the screening of mental impairments in routine health examinations.

Psychological interventions could be included as an effective part of mental health improving interventions considered for patients with MUS. Therefore, a collaborative working relationship between physicians, psychologists and psychiatrists should be established to manage health care users with medically unexplained symptoms.

## Conclusion

In conclusion, our results revealed that some psychological problems might relate to vulnerability to somatic symptoms. Therefore, screening for psychological impairments can improve clinical outcomes. 

## Limitations

Several limitations should, however, be taken into account in interpreting our findings. It is possible that somatic manifestation of psychological problems is a cultural phenomenon (32). Therefore, our results should be generalized to other cultures and populations with caution.

 Lack of psychiatric interview was another limitation of this study, so it is recommended for future studies. Finally, the present study included the relatively small sample size, so conducting further studies with a larger sample size is highly recommended.

In spite of these limitations, our findings had some important implications for physicians in management of their patients. They should be aware of the relationship between mental health and somatic symptoms. 
